# Robot-Assisted Laparoscopic Partial Colpectomy and Intracorporeal Ileal Conduit Urinary Diversion (Bricker) for Cervical Adenocarcinoma Recurrence

**DOI:** 10.1155/2015/241094

**Published:** 2015-11-08

**Authors:** Jennifer Uzan, Caroline Cornou, Chérazade Bensaid, François Audenet, Charlotte Ngô, Anne-Sophie Bats, Fabrice Lecuru

**Affiliations:** ^1^Assistance Publique-Hôpitaux de Paris, Hôpital Européen Georges-Pompidou, Chirurgie Cancérologique Gynécologique et du Sein, 20-40 rue Leblanc, 75015 Paris, France; ^2^Assistance Publique-Hôpitaux de Paris, Hôpital Européen Georges-Pompidou, Chirurgie Urologique, 20-40 rue Leblanc, 75015 Paris, France; ^3^Sorbonne Paris Cité, Faculté de Médecine, Université Paris Descartes, 15 rue de l'Ecole de Médecine, 75006 Paris, France; ^4^INSERM UMR-S 1124, Université Paris Descartes, 45 rue des Saints-Pères, 75006 Paris, France

## Abstract

Ileal conduit urinary diversion (Bricker) is a standard surgical open procedure. The Da Vinci robot allowed precision for this surgical procedure, especially for intracorporeal suturing. Meanwhile, few reports of robot-assisted laparoscopic ileal conduit diversion (Bricker) are described in the literature. We report the case of a 69-year-old patient with a vaginal recurrence of cervical adenocarcinoma associated with vesicovaginal fistula treated by robot-assisted laparoscopic partial colpectomy and ileal conduit urinary diversion (Bricker). The robot-assisted laparoscopic procedure followed all surgical steps of the open procedure. Postoperative period was free of complications.

## 1. Presentation of the Case

A 69-year-old patient without previous surgical history was referred to our center with a diagnosis of high-grade endocervical adenocarcinoma. Initial Magnetic Resonance Imagery (MRI) found an endocervical tumor measuring 65*∗*30 mm, with an extension to the exocervix, third upper vagina, and proximal parametria (FIGO stage IIB). No metastasis or adenomegaly was found on the CT-scan. The staging para-aortic lymphadenectomy retrieved eight lymph nodes that were free of disease. She received concomitant 3D intensity modulated radiotherapy treatment (45 Grays on cervix and uterus, 45 Grays on common, external, and internal iliac chain) and 6 cycles of platinum based chemotherapy (68 mg of cisplatin per cycle) followed by endobrachytherapy with iridium 192 (15 Grays). Then, she had total hysterectomy and bilateral salpingo-oophorectomy. The final pathology report showed residual adenocarcinoma with lymphovascular space involvement and vaginal margins free of disease. The six-month follow-up MRI found a recurrent disease of 20*∗*17 mm located in the right vaginal cul-de-sac and the PET-CT did not show any metastasis. A robot-assisted laparoscopic partial colpectomy was performed to treat this recurrence. During the surgery, it was noted that the bladder was fragilized because of radiation and past surgeries. An injury in the posterior surface of the bladder occurred and required a 2/0 Vicryl sutures; then, a urinary catheter was placed in the bladder. The final pathology report confirmed a recurrent high-grade endocervical adenocarcinoma with positive surgical margins. Two weeks after the colpectomy, the patient had rectal tenesmus and bladder pain; pelvic MRI showed a vesicovaginal fistula. Endorectal echoendoscopy was normal and PET-CT did not show any metastasis. The multidisciplinary oncologic committee proposed an anterior exenteration with Bricker urinary diversion to treat the fistula and the recurrence of endocervical cancer after colpectomy with positive margins. In order to decrease surgical morbidities for this fragile patient, we proposed a robot-assisted laparoscopy in this indication.

## 2. Technique

The localization of the definitive stoma was determined the day before surgery on a standing and sitting position of the patient. The stoma should be placed on the right lower quadrant of the abdomen distant from the bone relief of the iliac crest and from a skinfold in order to enable easy equipment. No digestive preparation was needed the days before.

Surgical procedure was made under general anesthesia. The patient was placed supine with legs on spreader bars. The port placement consisted with the camera placed through the umbilicus in a transperitoneal fashion and three 8 mm ports for the robotic arms placed 9 cm apart, with the fourth arm on the patient's left side. An additional 12 mm port was placed in the left lower quadrant and another additional 5 mm port was placed on the left upper quadrant for the bedside assistant.

### 2.1. Anterior Pelvectomy

**Figure 1:**
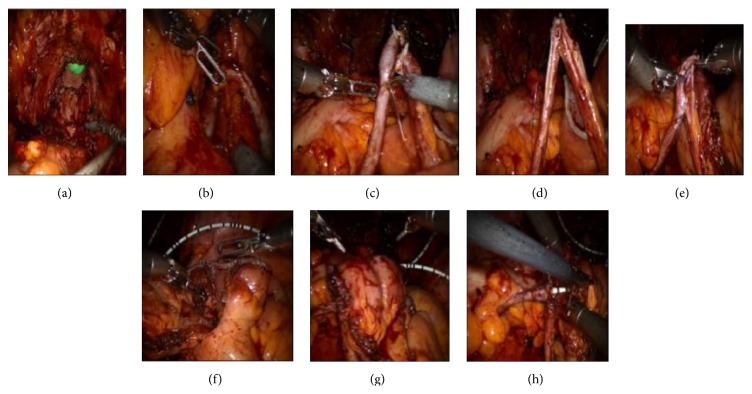
Per procedure views, (a) final pelvic view after cystectomy and partial colpectomy; (b) left ureter transferred to the right side behind the sigmoid mesocolon; (c) ureters that were recut; (d) ureters that were spatulated over 2 cm; (e) ureters that were sutured together with a 5-0 PDS running suture; (f) side-to-side anastomosis of the small bowel; (g) Wallace ureteroileal 5-0 PDS anastomosis; (h) 8 French ureteral stents that were placed in each ureter.

Primarily, postoperative adherence was liberated using bipolar energy and then the peritoneum lateral to the sigmoid colon was incised to allow access to the left iliac vessels and left ureter. The same gestures on the right side allowed access to the right iliac vessels and right ureter. Secondarily, both ureters were dissected from their lumbar portion to their bladder insertion. Their distal portion was clipped with Hemolocks, divided at its insertion into the bladder and tagged with 5 cm 3-0 Vicryl suture. Then, the prerectal and posterior vesical plan was developed. Rectovaginal wall was dissected in order to individualize the vagina and carry the total colpectomy. The superior and inferior vesical vessels, including the umbilical and uterine arteries, were divided with thermofusion ligation. The urachus was mobilized, dividing the medial and median umbilical ligament as far as possible proximally. The peritoneal incision and the dissection were conducted laterally to the medial umbilical ligament, in the Retzius space. Then, access for the apical dissection was obtained after bilateral incision of the endopelvic fascia. The pubourethral suspensory ligaments were identified and dissected, allowing the urethra and bladder to drop inferiorly. The urethra was then dissected from under the dorsal vein complex so that the only remaining attachments of the specimen were the urethral meatus and a small portion of the vagina. The urethra was divided and the final specimen was extracted by vaginal route before the vagina was closed.  Figure 1(a) shows final pelvic view after cystectomy and total colpectomy.

### 2.2. Total Intracorporeal Robot-Assisted Ileal Conduit (Bricker) Urinary Diversion

The first step consisted in the retroperitoneal transfer of the left ureter to the right side behind the sigmoid mesocolon, anterior to the sacral promontory, thanks to the 3-0 Vicryl suture tagged on it (Figure 1(b)). Then both ureters were presented by the robotic fourth arm in order to be cut with scissors over 1 cm distally (Figure 1(c)), spatulated over 2 cm (Figure 1(d)), and finally sutured together using a 5-0 PDS laterolateral running suture (Figure 1(e)).

The second step consisted in preparation of the ileal loop (Bricker). A 10 cm ileal segment was identified 20 cm away from to the ileocecal valve and exposed with two transparietal sutures of Monocryl 3/0. The ileal segment was isolated using staplers, Endo GIA Universal, 60-3.5, Titanium by Covidien. The small bowel continuity was stapled by side-to-side ileoileal anastomosis using staplers, Endo GIA Universal, 60-3.5, Titanium by Covidien, through the port of the fourth robotic arm after dedocking (Figure 1(f)). The defect in the mesentery was closed using running 3-0 Vicryl suture.

The third step consisted in Wallace 1 anastomosis. The proximal part of the Bricker was opened with scissors and Wallace 1 ureteroileal anastomosis was made using 5-0 PDS (Figure 1(g)). The anastomosis was protected by two 8-French mono-J ureteral stents introduced through the 12 mm port. The distal extremity of the Bricker was opened allowing the bedside assistant to put the stents with a grasper through the ileal conduit and then in each ureter using a hydrophilic wire (Figure 1(h)). The correct position of the stents was verified by injection of saline and they were fixed to each ureter with a stich of Vicryl Rapide 4-0. Cutting the left one slantwise and the right one straight differentiated 8 F ureteral stents outwardly. Once the stents were in place, Wallace 1 anastomosis could be achieved. The distal end of the ileal conduit was then externalized through the predetermined stoma and port site in the right lower quadrant of the abdomen and sutured using absorbable sutures of Vicryl 4.0.

**Figure 2:**
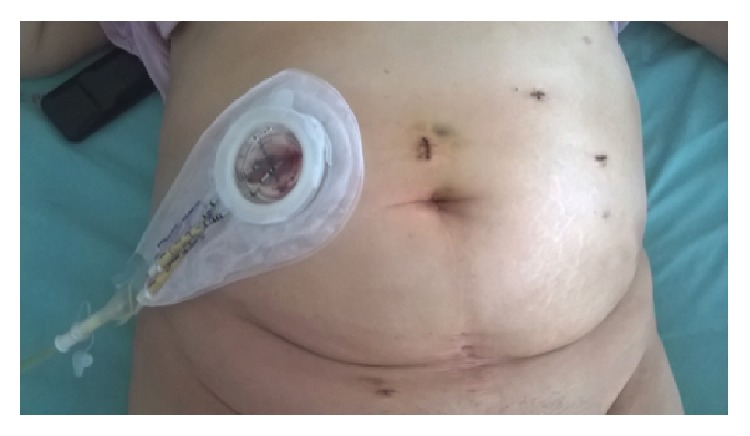
Postoperative patient's abdomen.

Postoperative patient's abdomen was shown in Figure 2.

## 3. Outcome

Operating time was 570 minutes. Injuries of the venous plexus of Retzius during cystectomy were responsible for blood loss requiring blood transfusion; however, blood loss during Bricker procedure was negligible.

Postoperative period was free of complications. The patient never had fever.

Diuresis was superior to 2 liters per day and blood electrolytes and creatinine remained stable. A clear liquid diet was started on postoperative day 2 and the patient recovered intestinal transit on postoperative day 7.

Ureteral stents were removed without complications on postoperative days 11 and 12 after bacteriologic exam on each ureteral stent.

The patient returned to normal activity within 2 weeks from date of surgery.

Final pathology exam found adenocarcinoma on the bladder and on the vagina. The margins were free of disease. No adjuvant treatment was required.

Seven months after the surgery, the patient was free of recurrence and had totally recovered from surgery.

## 4. Discussion

Total intracorporeal robot-assisted ileal conduit (Bricker) urinary diversion has been described either alone for prostatocutaneous fistula [1] or associated with radical cystoprostatectomy for cancer [2–4]. All operative steps of the standard open procedure were replicated in the robot-assisted procedure without open conversion.

In our case, operative time duration including cystectomy, colpectomy, and ileal conduit urinary diversion (Bricker) was 570 minutes. In the literature, mean operative time for robot-assisted procedure varied between 600 minutes for ileal conduit urinary diversion (Bricker) alone [1] and 456 [264–780] [4] to 828 minutes when associated with radical cystoprostatectomy [2]. Mean estimated blood loss varied between 50 and 2200 mL in the literature [2–4].

In our case, short-term complication was functional ileus. Functional ileus is the more frequent short-term complication of the standard open procedure [5] and laparoscopic procedure [6]. Other short-term complications of the standard procedure appeared to be gastrointestinal, including ileus, fistula, and evisceration. Long term complications of the standard procedure appeared to be parietal or urological including peristomial hernia, acute pyelonephritis, ureteroileal stricture, or urinary stones [5]. In a retrospective study of laparoscopic Bricker for neurogenic bladder, functional ileus was the major short-term complication associated with ureteroileal anastomosis stricture after removing the ureteral stents with Bricker technique anastomosis. However, no long term complications occurred during five years of follow-up: intravenous urography revealed prompt, symmetric renal function, preservation of renal parenchyma, and absence of reflux [6].

In comparison with open procedure, robot-assisted procedure seems to provide perioperative benefits, such as reduced surgical morbidities, and better functional outcome, such as early recovery, excellent cosmetic results, and increased quality of life. Nonetheless, the practical application of the robot-assisted procedure requires improved long-term outcomes compared to open procedure. Also, a cost benefit study would be interesting to compare the robot-assisted and standard open procedures.
